# Supplementary material for: Non-invasive cancer detection in canine urine through Caenorhabditis elegans chemotaxis
Source: Front Vet Sci. 2022 Aug 9;9:932474. doi: 10.3389/fvets.2022.932474 (PMC9396970; doi:10.3389/fvets.2022.932474)
Supplement: Supplementary file 1 [file Data_Sheet_1.DOCX]

Supplementary Material

# Supplementary Figures

**
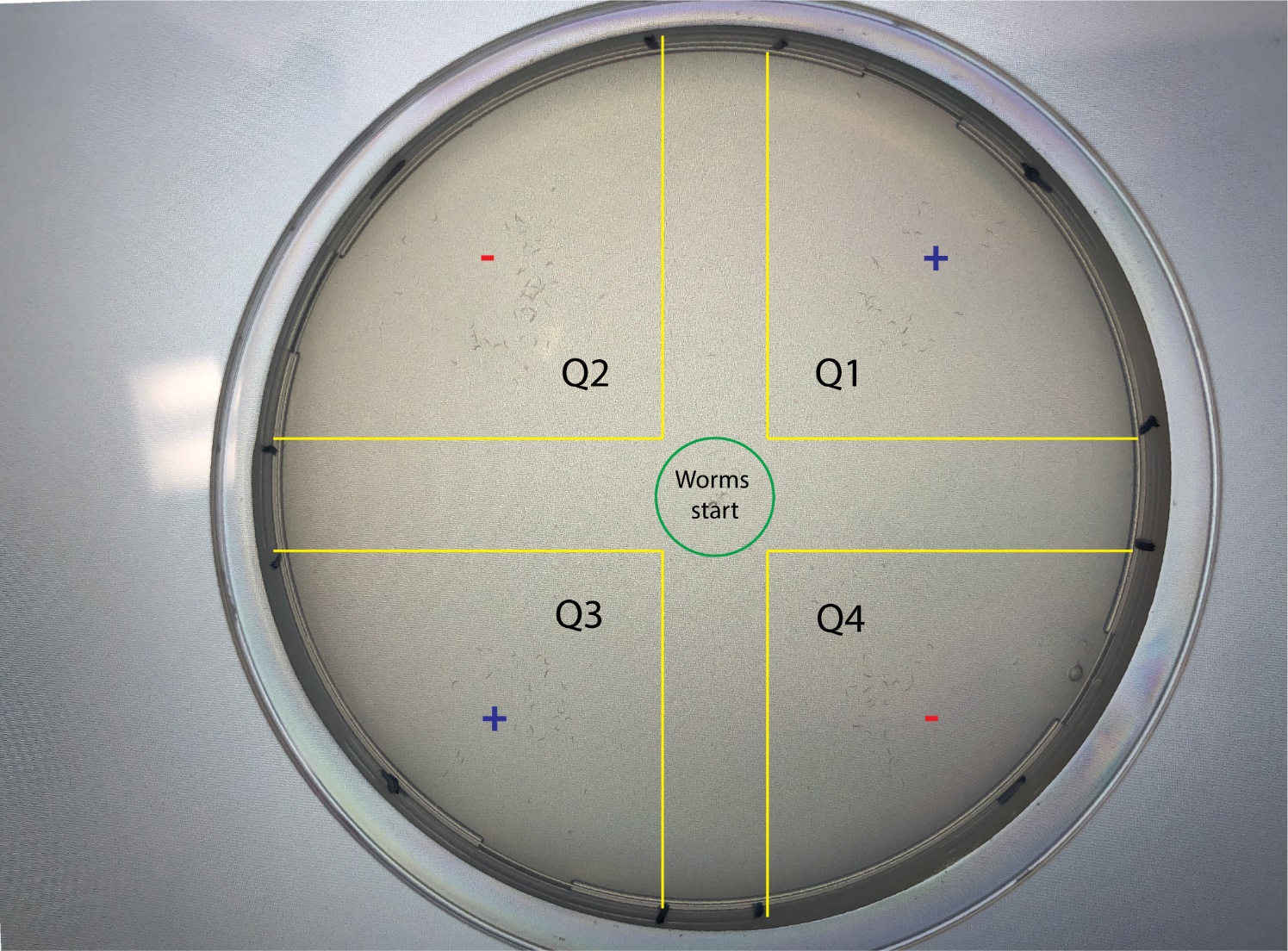
**

**Supplementary Figure 1.** Each assay plate is divided into four quadrants. The positive quadrants (Q1, Q3) are marked with a “plus” sign, which designates the location of the tested urine sample. The negative quadrants are marked with a “minus” sign, which designates the location of the control buffer. *C. elegans* are placed at the center of the green circle at the beginning of the assay. After the assay is complete, animals are counted in each quadrant within the bounds of the yellow lines to avoid counting those that are close to the quadrant borders or that remain at the center of the plate.


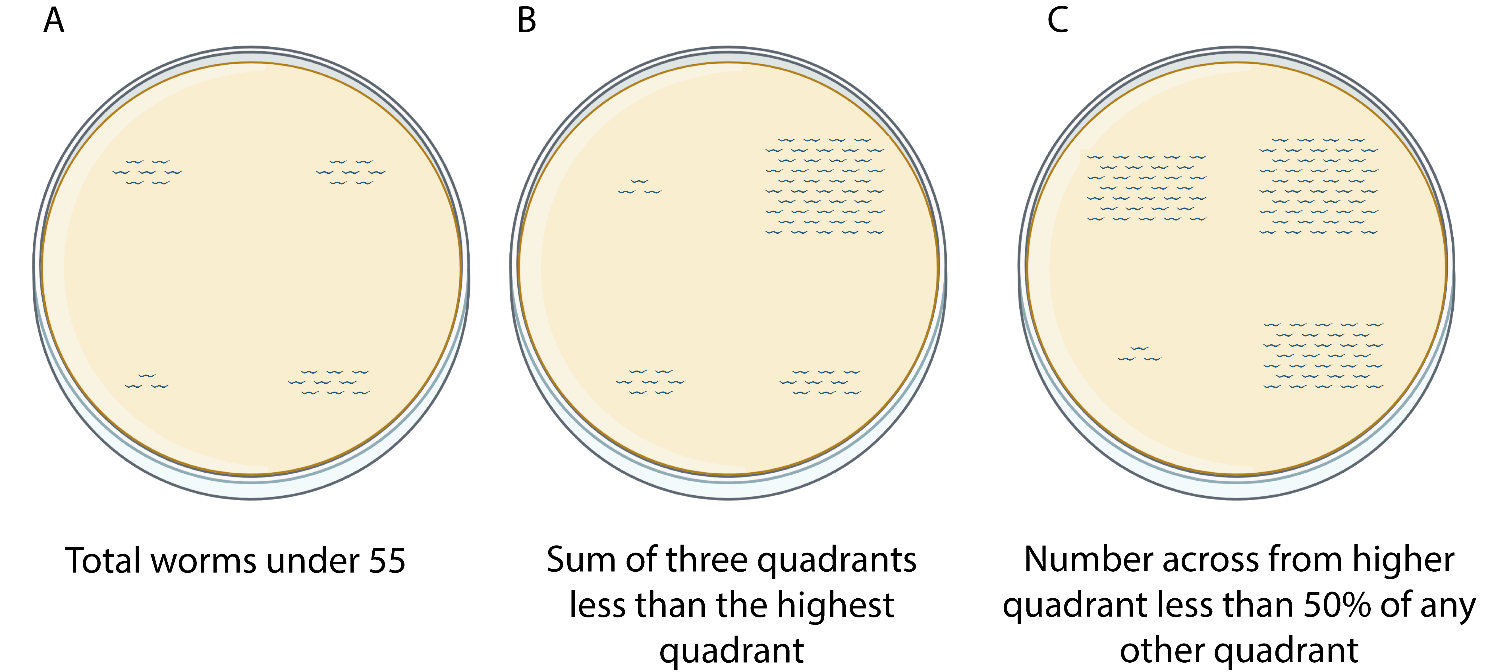


**Supplementary Figure 2.** Examples of conditions in which plates are discarded. **A** The total number of animals on the plate is less than 55:

$$Q1+Q2+Q3+Q4\leq55$$

**B** The sum of the total animals in three quadrants is less than the total number of animals in the fourth quadrant, e.g.

$$Q1\geq Q2+Q3+Q4$$

**C** The number of animals across from the quadrant with the highest total has 50% or less than the number of animals on any other quadrant, e.g.

$$Q1\geq Q2\left| Q3 \right|Q4, Q3\leq\frac{Q1}{2}\left| \frac{Q2}{2} \right|\frac{Q4}{2}$$

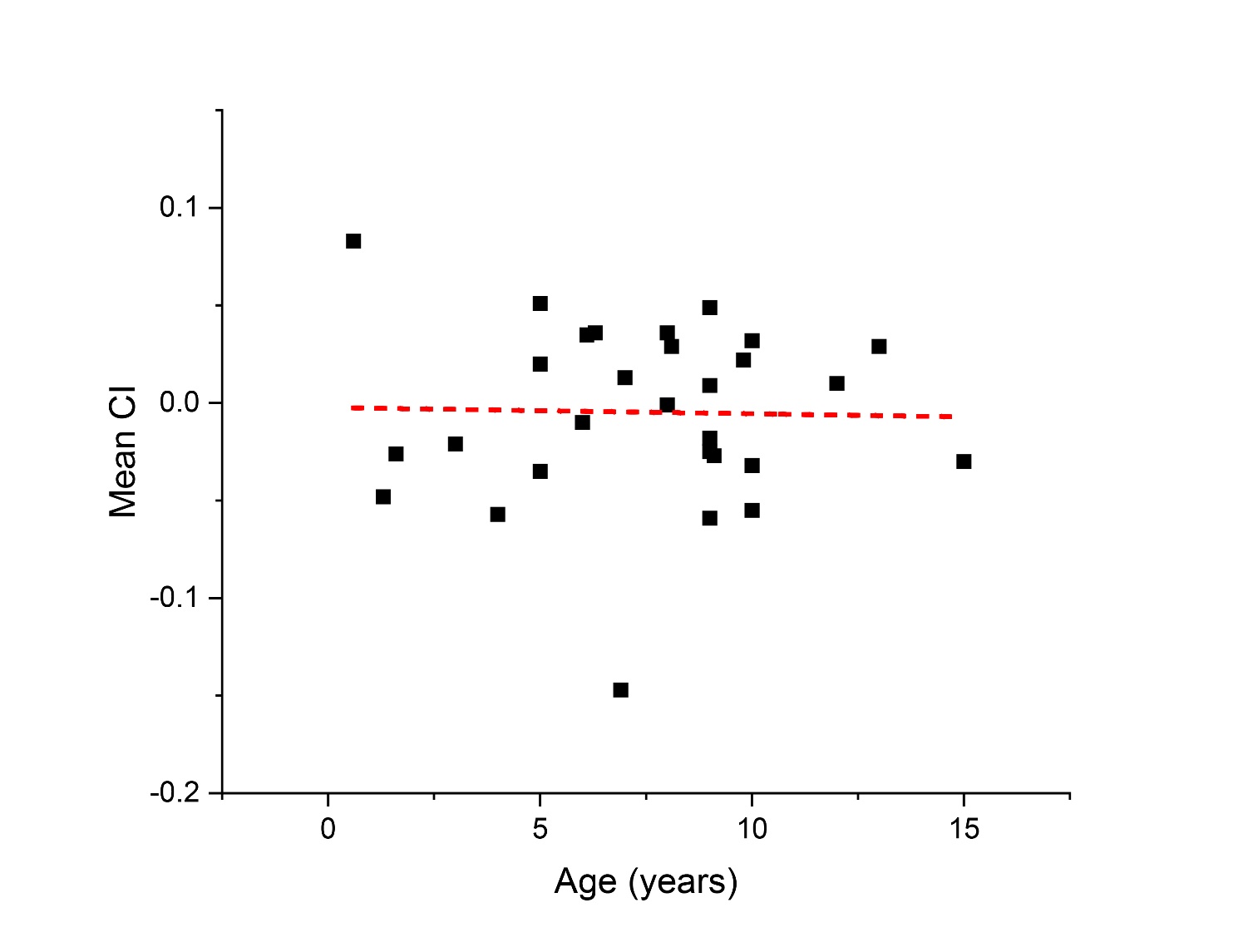


**Supplementary Figure 3.** No correlation was observed for the observed chemotaxis index relative to age. Each data point represents the mean CI observed for each animal. Red dotted line is the linear best fit for the data (Slope=3.17x10^-4^).

# Supplementary Tables

**Supplementary Table 1.** CI replicates for initial set of cancer and non-cancer assays. The CI for each non-discarded replicate is listed beneath the code of each cancer and non-cancer patient.

**Supplementary Table 2.** Chemotaxis data summary for initial cancer and non-cancer data comparison. M=male, not castrated. F=female, not spayed. CM=male, castrated. SF=female, spayed. Unlisted gender information indicates incomplete information on castration or spaying.

**Supplementary Table 3.** Sample assay repeats for dog patients with (P03) and without (P09) diagnosed cancer. Each of the assay repeats shows a mean CI above and below the moderate risk threshold for cancer and non-cancer patients, respectively.

|  | P03 (Mast Cell Tumor) | | | P09 (Non-cancer) | | |
| --- | --- | --- | --- | --- | --- | --- |
|  | Assay 1 | Assay 2 | Assay 3 | Assay 1 | Assay 2 | Assay 3 |
| Replicate 1 | 0.123 | 0.223 | 0.109 | -0.159 | -0.051 | 0.294 |
| Replicate 2 | 0.233 | 0.278 | 0.052 | -0.153 | 0.163 | -0.294 |
| Replicate 3 | 0.12 | -0.042 | 0.045 | -0.167 | -0.136 | -0.127 |
| Replicate 4 | 0.174 | 0.286 | 0.071 | 0.031 | 0.164 | 0.147 |
| Replicate 5 | 0.102 | 0.147 | 0.211 | 0.096 | -0.31 | -0.143 |
| Replicate 6 | 0.082 |  | 0.027 | 0.056 |  |  |
| Replicate 7 | 0.109 |  |  | 0.053 |  |  |
|  |  |  |  |  |  |  |
| Mean CI | 0.135 | 0.178 | 0.086 | -0.035 | -0.034 | -0.025 |

**Supplementary Table 4.** CI replicates for ten samples of lymphoma, mast cell tumor, melanoma, and hemangiosarcoma. The CI for each non-discarded replicate is listed beneath the code of each cancer patient.

**Supplementary Table 5.** Chemotaxis data summary for ten samples of lymphoma, mast cell tumor, melanoma, and hemangiosarcoma. M=male, not castrated. F=female, not spayed. CM=male, castrated. SF=female, spayed. Unlisted gender information indicates incomplete information on castration or spaying.

**Supplementary Table 6.** CI replicates for sixteen additional samples without a confirmed cancer diagnosis. The CI for each non-discarded replicate is listed beneath the code of each cancer patient.

**Supplementary Table 7.** Chemotaxis data summary sixteen additional samples without a confirmed cancer diagnosis. M=male, not castrated. F=female, not spayed. CM=male, castrated. SF=female, spayed. Unlisted gender information indicates incomplete information on castration or spaying.
